# Different subtypes of EGFR exon19 mutation can affect prognosis of patients with non-small cell lung adenocarcinoma

**DOI:** 10.1371/journal.pone.0201682

**Published:** 2018-11-01

**Authors:** Yingying Tian, Jiuzhou Zhao, Pengfei Ren, Bo Wang, Chengzhi Zhao, Chao Shi, Bing Wei, Jie Ma, Yongjun Guo

**Affiliations:** 1 Department of Molecular Pathology, Henan Cancer Hospital, The Affiliated Cancer Hospital, Zhengzhou University, Zhengzhou, China; 2 School of Pharmaceutical Sciences, Zhengzhou University, Zhengzhou, China; Wayne State University School of Medicine, UNITED STATES

## Abstract

**Aims:**

In this study, we determined whether different subtypes of epidermal growth factor receptor (EGFR) exon19 mutation are associated with the therapeutic effect of EGFR-tyrosine kinase inhibitors (TKIs) on advanced non-small cell lung adenocarcinoma.

**Methods:**

A total of 122 patients with stage III or IV non-small cell lung adenocarcinoma were retrospectively reviewed. Clinical characteristics of these patients, including progression-free survival (PFS) outcome for EGFR-TKI treatment, were analyzed.

**Results:**

According to the mutation pattern, we classified the in-frame deletions detected on EGFR Exon19 into three different types: codon deletion (CD), with a deletion of one or more original codons; codon substitution and skipping (CSS), with a deletion of one or two nucleotides but the residues could be translated into a new amino acid without changing following amino acid sequence; CD or CSS plus single nucleotide variant (SNV) (CD/CSS+SNV), exclude CD or CSS, there’s another SNV nearby the deletion region. The clinical characteristics of three groups were analyzed and as a result, no significant difference was found. By comparing the average number of missing bases and amino acids of the three mutation subtypes, it could be discovered that the number of missing bases and amino acids of the three mutation subtypes is diverse, and group CSS> group CD> group CD/CSS+SNV. Finally, survival analysis was performed between three groups of patients. The median PFS of group CD, group CSS and group CD/CSS+SNV was 11 months, 9 months and 14 months respectively. There was a distinct difference in the PFS between group CSS and group CD/CSS+SNV (P = 0.035<0.05), and the PFS of group CD/CSS+SNV was longer.

**Conclusions:**

Different mutation subtypes of EGFR exon19 can predict the therapeutic effect of EGFR-TKIs on advanced non-small cell lung adenocarcinoma.

## Introduction

Lung cancer is the leading cause of cancer-related death worldwide[1]. Non-small cell lung cancer (NSCLC) accounts for about 85% of lung cancer[2, 3]. With the emergence of EGFR-TKIs, such as gefitinib, erlotinib, and icotinib, the survival time and life quality of patients with lung cancer have been obviously improved [4–6]. Provided the inconsistent effect of EGFR-TKIs on different patients, some patients received EGFR-TKIs with apparent efficacy, while others did not work. Therefore, it is particularly important to screen out these patients who showing a better response to EGFR-TKIs.

Epidermal growth factor (EGF) and its receptor (EGFR), which is most closely related to lung adenocarcinoma, were discovered by Stanley Cohen of Vanderbilt university in 1986[7]. Human EGFR gene is located on chromosome 7p11.2, which contains 28 exons, sizing about 200kb. EGFR exon 18 ~ 24 encodes tyrosine kinase functional region of receptor. The vast majority of EGFR mutation occur in NSCLC, especially in non-smoking Asian women[8–10]. EGFR exon19 mutation can increase the kinase activity of EGFR, leading to the hyperactivation of downstream pro-survival pathways, and consequently confer oncogenic properties on EGFR[11]. Compared to patients with negative EGFR mutation, EGFR-mutated patients showed longer PFS (11.5 months)[12]and overall survival(OS) (15.4 months)[13]. The common subtypes of EGFR mutation include exon19 deletion (19del) and point mutation on exon21(L858R), accounting for 33.1% and 40.9% respectively[14]. Multiple randomized clinical trials have demonstrated that EGFR 19del and L858R are highly correlated with sensitivity to EGFR-TKI in NSCLC, especially 19del[15, 16]. However, Patients with EGFR 19 del results different PFS after EGFR TKI treatment, and the reason is still not clear. This retrospective study reviewed the medical records of EGFR exon19 mutant advanced NSCLC patients undergoing EGFR-TKIs treatment, so as to evaluate the association of different subtypes of exon19 mutation with EGFR-TKI efficacy in EGFR-mutant advanced non-small cell lung adenocarcinoma patients.

## Materials and methods

### Ethical statement

This research involving human participants have been approved by the Henan Cancer Hospital Institutional Review Board (IRB), and all clinical investigation have been conducted according to the principles expressed in the Declaration of Helsinki. The ethical review number is 2018123.

### Patients

Inclusion criteria were as follows: (1) patients with pathologically confirmed non-small cell lung adenocarcinoma who underwent EGFR mutation screening and TKIs treatment at Henan Cancer Hospital between 2015 and 2017;(2) patients who merely harbored EGFR exon19 mutation and did not receive other treatment before targeted therapy. A total of 122 patients were collected between 2015 and 2017 in Henan Cancer Hospital. EGFR exon19 mutation was detected in tumor tissues by the method of Next Generation Sequencing (NGS). All subjects were administered with gefitinib(n = 76), erlotinib (n = 10), or icotinib (n = 36), until disease progression or intolerance to adverse events. Since the above three EGFR-TKIs have similar therapeutic effect[17], this study did not take into account the different impact of targeted drugs such as gefitinib, erlotinib and icotinib on patients' PFS.

### Detection of EGFR exon19 mutation by NGS

gDNA from formalin-fixed paraffin-embedded (FFPE) tissues was extracted using a QIAamp Circulating Nucleic Acid kit (Qiagen, Hilden, Germany), according to the manufacturer’s recommendations. All gDNA samples were first assessed using a NanoDrop-2000(Thermo Scientific, Wilmington, DE, USA), and then qualified by a Qubit 2.0 Fluorometer (Invitrogen, Carlsbad, CA, USA) using a Qubit dsDNA HS Assay Kit. gDNA was fragmented to about 200bp with Diagenode SA. Libraries were constructed using a human polygenic mutation detection kit (Burning Rock Dx), which analyzes target regions of 8 genes (EGFR, KRAS, BRAF, ERBB2, ALK, ROS1, RET, and MET). The library size was checked using the Agilent High Sensitivity DNA Kit by the Bioanalyzer 2100 instrument (Agilent Technologies), and library concentration was evaluated with a Qubit 2.0 Fluorometer using the Qubit dsDNA HS Assay Kit, following the manufacturer’s instructions. Finally, sequencing was performed on the Illumina NextSeq 550.

### Follow up

All 122 patients were followed up through hospital medical records until disease progression or April 2018. In this study, response was classified by standard Response Evaluation Criteria in Solid Tumors (RECIST). Confirmed by imaging, the total maximum diameter of the target lesion increased by at least 20%, or new lesion appeared, which was defined as disease progression. PFS was defined as the duration from administration of EGFR-TKIs to disease progression. The object of study had a median follow-up period of 8 months (range, 1 to 53 months), with a 100% follow-up seen. During the follow-up period, 82 patients have been observed with progression.

### Statistics

All statistical analyses were conducted using the statistical software SPSS version 16.0. Univariate analyses were performed with a log-rank test. Clinical characteristics of the different groups, including gender, age, smoking history, drinking history, family history and Eastern Cooperative Oncology Group Performance Status (ECOG PS) were compared by χ2 test or Fisher exact test, and the survival was estimated with the Kaplan-Meier method. All P values were 2-sided and P<0.05 was considered statistically significant.

## Results

### Clinical characteristics of patients with EGFR exon19 mutation

A total of 122 lung adenocarcinoma patients with EGFR exon19 mutation were enrolled in this study. The participants included 55 men and 67 women, they had a median age of 57 years (range, 27 to 81 years); 100 cases at ages of 67 years or less, 22 cases aged over 67 years. Of the total study subjects, 30 cases were identified with history of smoking or alcohol drinking while 92 cases were neither smokers nor drinkers; 25 patients were discovered with family history of cancer and 97 patients were not. The ECOG score of 108 cases was 0, 14 cases were 1. According to the TNM staging system for NSCLC[18], 14 patients were identified as staged III, and 108 patients were staged IV. 122 patients had not received any other therapies before EGFR-TKIs treatment. The clinical characteristics of 122 patients were analyzed by univariate analysis, it was found that mentioned clinical data did not affect the survival of patients with EGFR exon19 mutation (all P values > 0.05) (Table 1).

**Table 1:** Univariate analysis of 122 patients with EGFR exon19 mutation.

Characteristics	No. of patients N = 122(%)	Median PFS(months,95%CI)	*P*
**Gender**			0.612
Male	55(45.1)	13(12.089–13.911)	
Female	67(54.9)	10(8.481–11.519)	
**Age(years)**			0.12
≤67	100(82)	12(10.318–13.682)	
>67	22(18)	9(4.361–13.639)	
**Smoking**			0.708
Yes	30(24.6)	12(7.387–16.613)	
No	92(75.4)	11(9.376–12.624)	
**Drinking**			0.894
Yes	30(24.6)	13(11.961–14.039)	
No	92(75.4)	11(9.384–12.616)	
**Family history**			0.332
Yes	25(20.5)	12(8.342–15.658)	
No	97(79.5)	11(9.173–12.827)	
**ECOG PS**			0.132
0	108(88.5)	12(10.42–13.58)	
1	14(11.5)	10(3.781–16.219)	

PFS, progression-free survival; CI, confidence interval; ECOG PS, eastern cooperative oncology group performance status.

### Distribution of EGFR exon19 mutation subtypes in NSCLC

**Fig 1:**
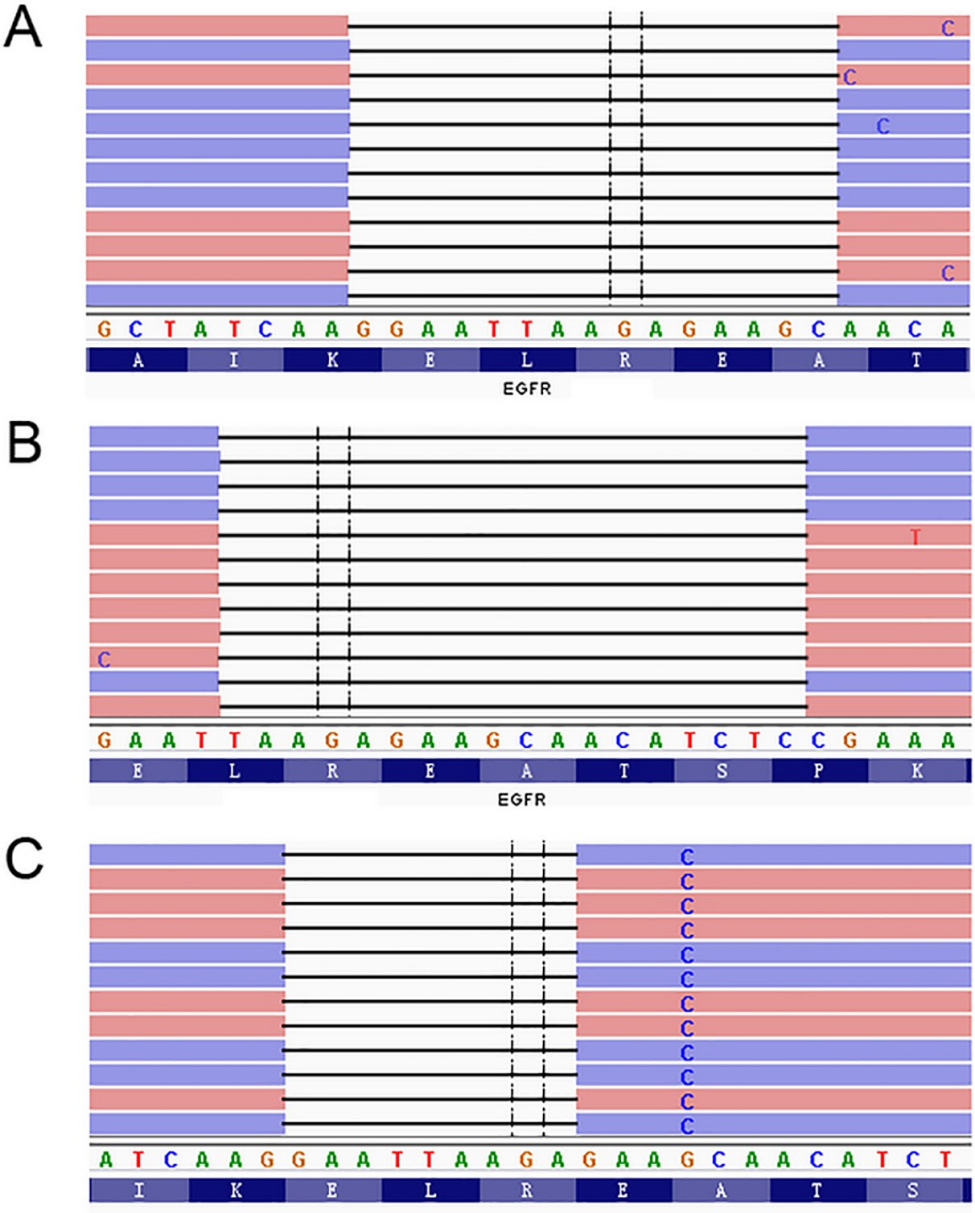
Three mutation subtypes of EGFR exon19. The mutation pattern of group CD(codon deletion), with a deletion of one or more original codons(A); The mutation pattern of group CSS(codon substitution and skipping), with a deletion of one or two nucleotides but the residues could be translated into a new amino acid without changing following amino acid sequence(B); The mutation pattern of group CD/CSS+SNV(codon deletion or codon substitution and skipping plus SNV), exclude CD or CSS, there’s another SNV nearby the deletion region(C).

There were 16 mutation subtypes in this study, including c.2235_2249del, c.2236_2250del, c.2239_2247del, c.2240_2254del, c.2237_2251del, c.2237_2254del, c.2240_2257del, c.2235_2251delins, c.2235_2252delins, c.2236_2248delins, c.2236_2255delins, c.2237_2255delins, c.2237_2257delins, c.2239_2248delins, c.2239_2251delins, c.2252_2276delins, respectively (Table 2). In this study, we divided all patients into three groups based on EGFR exon19 mutation pattern: group CD, group CSS and group CD/CSS+SNV (Fig 1). The clinical data of three groups were analyzed by χ^2^ test. It was discovered that there was no significant difference in the clinical data between three groups (Table 3). Furthermore, Independent- Samples T Test was conducted for comparing the number of missing bases and missing amino acids of different mutation types. The mean number of missing bases in group CD was 14.93, 17.2 in group CSS and 14.18 in group CD/CSS+SNV. The results showed significant differences in the number of missing bases between group CD and group CD/CSS+SNV, likewise in group CSS and group CD/CSS+SNV (P<0.05). The mean number of missing amino acids in group CD was 4.98, 5.73 in group CSS and 4.76 in group CD/CSS+SNV. Consistently, there were also significant differences in the number of missing amino acids between group CD and group CSS, similarly in group CSS and group CD/CSS+SNV (P<0.05) (Table 4).

**Table 2:** Mutation subtypes of 122 EGFR exon19 mutation.

Base change	Amino acids change	N(%)
c.2235_2249del	p.Glu746_Ala750del	49(40.2)
c.2236_2250del	p.Glu746_Ala750del	32(26.2)
c.2239_2247del	p.Leu747_Glu749del	1(0.8)
c.2240_2254del	p.Leu747_Thr751del	8(6.6)
c.2237_2251del	p.Glu746_Thr751delins	4(3.3)
c.2237_2254del	p.Glu746_Ser752delins	1(0.8)
c.2240_2257del	p.Leu747_Pro753delins	10(8.2)
c.2235_2251delins	p.Glu746_Thr751delins	1(0.8)
c.2235_2252delins	p.Glu746_Thr751delins	1(0.8)
c.2236_2248delins	p.Glu746_Ala750delins	1(0.8)
c.2236_2255delins	p.Glu746_Ser752delins	1(0.8)
c.2237_2255delins	p.Glu746_Ser752delins	4(3.3)
c.2237_2257delins	p.Glu746_Pro753delins	1(0.8)
c.2239_2248delins	p.Leu747_Ala750delins	5(4.2)
c.2239_2251delins	p.Leu747_Thr751delins	2(1.6)
c.2252_2276delins	p.Thr751_Ile759delins	1(0.8)

**Table 3:** Clinical characteristics of three groups.

Characteristics	Total N = 122 n(%)	CD N = 90 n(%)	CSS N = 15 n(%)	CD/CSS+SNV N = 17 n(%)	*χ*^*2*^	*P*
**Gender**					1.134	0.567
Male	55(45.1)	38(42.2)	8(53.3)	9(52.9)		
Female	67(54.9)	52(57.8)	7(46.7)	8(47.1)		
**Age(years)**					1.123	0.57
<67	99(81.1)	75(83.3)	11(73.3)	13(76.5)		
≥67	23(18.9)	15(16.7)	4(26.7)	4(23.5)		
**Smoking**					0.226	0.893
Yes	30(24.6)	23(25.6)	3(20)	4(23.5)		
No	92(75.4)	67(74.4)	12(80)	13(76.5)		
**Drinking**					0.522	0.77
Yes	30(24.6)	23(25.6)	4(26.7)	3(17.6)		
No	92(75.4)	67(74.4)	11(73.3)	14(82.4)		
**Family history**					0.449	0.799
Yes	25(20.5)	18(20)	4(26.7)	3(17.6)		
No	97(79.5)	72(80)	11(73.3)	14(82.4)		
**ECOG PS**					0.89	0.641
0	109(89.3)	79(87.8)	14(93.3)	16(94.1)		
1	13(10.7)	11(12.2)	1(6.7)	1(5.9)		

CD, codon deletion; CSS, codon substitution and skipping; CD/CSS+SNV, CD or CSS plus SNV; ECOG PS, eastern cooperative oncology group performance status.

**Table 4:** The number of missing bases and amino acids of three groups.

Characteristics	CD Mean	CSS Mean	*P*	CD Mean	CD/CSS+SNV Mean	*P*	CSS Mean	CD/CSS+SNV Mean	*P*
**Missing base number**	14.93	17.2	<0.01	14.93	14.18	0.548	17.2	14.18	0.029
**Missing amino acid number**	4.98	5.73	<0.01	4.98	4.76	0.572	5.73	4.76	0.022

CD, codon deletion; CSS, codon substitution and skipping; CD/CSS+SNV, CD or CSS plus SNV

### Impact of EGFR exon19 mutation subtypes on prognosis of patients

**Fig 2:**
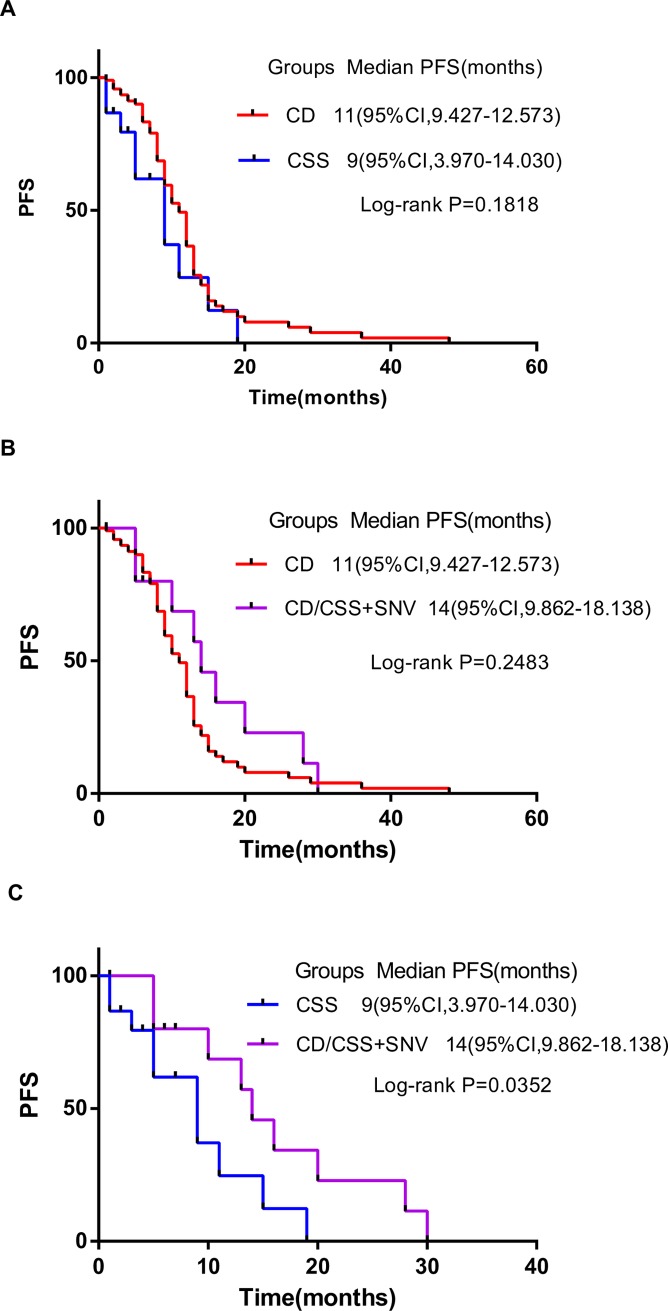
Kaplan-Meier curves of progression-free survival (PFS). Survival curves showing that there was no significant difference in PFS between group CD and CSS(A), between group CD and CD/CSS+SNV(B). Survival curves showing significant differences were found in PFS between group CSS and CD/CSS+SNV(C). CD, codon deletion; CSS, codon substitution and skipping; CD/CSS+SNV, CD or CSS plus SNV.

There were 90 patients in group CD, of whom 59 cases had progress and 31 cases had no progress; 15 patients in group CSS, 10 cases of whom had progress, 5 cases of whom did not. Group CD/CSS+SNV included 17 patients, 10 cases of whom had progress, while 7 cases of whom were not. Furthermore, the kaplan-meier survival analysis was performed on three groups. The median PFS in group CD was 11 months, the 95% confidence interval was 9.427–12.573, the median PFS in group CSS was 9 months, the 95% confidence interval was 3.97–14.03, the median PFS in group CD/CSS+SNV was 14 months, and the 95% confidence interval was 9.862–18.138. It was found that there was a significant difference in PFS between group CSS and group CD/CSS+SNV (P<0.05). The median PFS of patients in group CD/CSS+SNV is significantly higher than that in group CD and group CSS (Fig 2), which indicates that different mutation subtypes of EGFR exon19 can influence the therapeutic effect of EGFR-TKIs on advanced non-small cell lung adenocarcinoma.

## Discussion

A total of eight genes were detected by NGS in our study, including EGFR, KRAS, BRAF, ERBB2, ALK, ROS1, RET and MET, and it is common-accepted that EGFR gene mutation accounted for the majority. Various randomized clinical trials have emphasized that EGFR 19del and 21L858R mutation in NSCLC are highly sensitive to EGFR-TKIs, especially 19del[19, 20]. Currently, due to the limitation of gene detection technology, few institutions have studied the effect of mutation subtypes of EGFR exon19 on the prognosis of disease. The molecular pathology department of Henan Cancer Hospital introduced the Next 550 sequencer, which has a sensitivity up to 1/1000 for gene sequencing, providing a useful tool for us to study the mutation subtypes of EGFR exon19. This study is a retrospective analysis of all patients with EGFR exon19 mutation in Henan Cancer Hospital from 2015 to 2017, which aims to guide patients with advanced lung cancer to obtain more efficient treatment.

Wei B[21] implicated that tumor genesis driven by different EGFR mutations were mechanistically different. Zhang C[22] showed that patients with mutation of T790M had poor prognosis. As we all know, PFS and OS for advanced NSCLC patients with an exon 19 mutation was considerably higher than those with other subtypes of EGFR mutations[23, 24]. However, due to the individual imparity in the PFS of patients with EGFR exon19 mutation, we tried to figure out these patients who would have a better response from EGFR-TKIs treatment. According to the different pattern of EGFR exon19 mutation, patients were divided into group CD, group CSS and group CD/CSS+SNV. the survival of three groups were analyzed and found that the median PFS of group CD was 11 months, while group CSS was 9 months, and group CD/CSS+SNV was 14 months. Obviously, group CD/CSS+SNV had a longer PFS. Although the survival curves of group CD and group CD/CSS+SNV, group CD and group CSS were not considerably different(P>0.05), it was not difficult to find that the PFS of most patients in group CD/CSS+SNV is higher than that in group CD, and the PFS of most patients in group CD is higher than that in group CSS. Independent-Samples T Test was conducted for the number of missing bases and missing amino acids of different mutation types. The results showed that the number of missing bases and amino acids in group CD/CSS+SNV was the lowest, and the impact of group CD/CSS+SNV on the genome sequence was minimal, which may be the main reason for the longer PFS in group CD/CSS+SNV. In the future, we will engage on the mechanisms of such significant difference.

Some researchers reported different sensitivity of EGFR-TKIs in patients with different exon 19 deletion subtypes, while the arguments were controversial. Su J et al. grouped EGFR exon19 mutations according to different nucleotide changes and divided them into 39 subtypes. They found that patients with non-LRE deletions had a relatively long median PFS, but it was not significantly different from that of those with deletions E746 or L747 (16.0, 11.6, and 14.1 months, respectively; P = 0.463)[25]. Sutiman N et al. categorized mutations occurring in exon 19 into five subtypes according to the number of deleted nucleotides. However, comparison of PFS among the five different exon 19 mutation subtypes did not reveal any significant differences (overall P = 0.161)[26]. Our study, however, was contrary to previous studies in the way of grouping the EGFR exon 19 mutation, and uncovered the PFS of group CD/CSS+SNV was longer(P = 0.035).The advantage of our grouping approach is that we consider not only alterations on nucleotide level but also amino acids. What’s more, all EGFR exon19 deletions can be classified explicitly.

Oncogenic mutations including EGFR mutations were detected by NGS in this study. However, NGS is a labor-intensive and time-consuming method requiring specialized and costly facilities. What’s more, the availability of tumor tissues is not always satisfactory. Majority of NSCLC patients are diagnosed with advanced stages. Performing surgery or biopsy to obtain tissues from these patients is often impractical. Encouragingly, ctDNA(circulating cell-free tumor DNA) provide a high degree of specificity to detect EGFR mutations compared to tissue DNA. Moreover, ctDNA is capable of monitoring disease progression during EGFR-TKI treatment. Most importantly, ctDNA is relatively easy to obtain from patients with advanced NSCLC[27]. We are inclined to adopt ctDNA EGFR mutation test to predict EGFR-TKIs treatment efficacy in the future.

Our study showed that EGFR-TKIs had the best therapeutic efficacy on the group CD/CSS+SNV, which had the longest PFS in patients with EGFR exon19 mutation. Thus, EGFR exon19 mutation subtypes should be considered when predicting prognosis. However, the response of group CSS to EGFR-TKIs was relatively poor, combination therapy could be considered. Wang H et al. found that the PFS of patients was closely related with the mutation abundance of the EGFR gene after treatment with EGFR-TKI. First-line treatment with EGFR-TKIs in patients with high EGFR mutation abundance achieved more benefits in terms of PFS, while 26.7% was the best cutoff value to separate the low and high EGFR mutant abundance[28]. Furthermore, we want to explore whether EGFR mutation subtypes abundance could be used as a predictor for the extent of the response to EGFR-TKI therapy. This study still has limitations. Firstly, the study results may be skewed by the numbered cases of EGFR exon19 mutation; Secondly, the study results may be also influenced because of the instability of retrospective analysis. More data would be needed in the future to confirm the findings. To sum up, our study screened out patients with EGFR exon19 mutation who had a better response to EGFR-TKIs, which contributes to guiding the clinical treatment of advanced non-small cell lung adenocarcinoma.

## Supporting information

S1 File The original data of this study. (XLSX)
